# A generative adversarial network-based abnormality detection using only normal images for model training with application to digital breast tomosynthesis

**DOI:** 10.1038/s41598-021-89626-1

**Published:** 2021-05-13

**Authors:** Albert Swiecicki, Nicholas Konz, Mateusz Buda, Maciej A. Mazurowski

**Affiliations:** 1 Department of Electrical and Computer Engineering, Duke University, Durham, NC USA; 2 Department of Radiology, Duke University, Durham, NC USA

**Keywords:** Breast cancer, Cancer imaging, Cancer screening, Software

## Abstract

Deep learning has shown tremendous potential in the task of object detection in images. However, a common challenge with this task is when only a limited number of images containing the object of interest are available. This is a particular issue in cancer screening, such as digital breast tomosynthesis (DBT), where less than 1% of cases contain cancer. In this study, we propose a method to train an inpainting generative adversarial network to be used for cancer detection using only images that do not contain cancer. During inference, we removed a part of the image and used the network to complete the removed part. A significant error in completing an image part was considered an indication that such location is unexpected and thus abnormal. A large dataset of DBT images used in this study was collected at Duke University. It consisted of 19,230 reconstructed volumes from 4348 patients. Cancerous masses and architectural distortions were marked with bounding boxes by radiologists. Our experiments showed that the locations containing cancer were associated with a notably higher completion error than the non-cancer locations (mean error ratio of 2.77). All data used in this study has been made publicly available by the authors.

## Introduction

Deep learning methods have been shown to be highly successful in the analysis of medical images^1^. However, typically a large amount of data is needed to train accurate models. The collection of a large numbers of cases is particularly challenging when attempting to work with rare diseases. In screening populations, the prevalence of some diseases can be as low as 1%, resulting in a large number of normal exams, yet very few exams depicting abnormalities. One of the domains where we can observe such low prevalence is mammography, imaging exams intended to detect breast cancer in otherwise healthy women. Based on^2^, only 9812 out of 1,682,504 screening mammograms examinations performed between 2007 and 2013 consisted of cancerous alternations, resulting in an approximately 0.6% ratio between positive and negative test results. The three-dimensional, more modern form of mammography, called digital breast tomosynthesis (DBT) may find a slightly larger number of cancers since it provides better lesion visibility when compared with analog mammography or full-field digital mammography (FFDM)^3^. However, the prevalence of abnormal results remains very low.

Such imbalance in the training dataset causes significant problems when training deep learning algorithms and has been shown to negatively affect model performance^4^. In detection tasks, training difficulty already arises from the very limited number of images that contain abnormalities, but as in the case of mammography, this is made even worse when combined with the fact that the abnormalities themselves occupy relatively small parts of the images. Therefore, in order to make some sort of meaningful training progress, it becomes crucial to effectively utilize images that do *not* contain abnormalities, which *are* available in abundance.

Current supervised deep learning-based detection algorithms are not well-designed to take advantage of images that do not contain abnormalities. Images without abnormalities are used in anomaly detection algorithms where models try to learn data distributions and, based on normal data, try to predict unusual behaviors. One of the approaches to utilizing images with no abnormalities is to extract feature representation from normal data before training models with rare abnormal data. The most popular ways of extracting features generally (1) use a compression-decompression network called an *autoencoder*^5,6^or (2) involve generative adversarial networks (GANs) to learn data distributions^7,8^.

Our hypothesis is that breasts, similarly to many other objects, have a certain expected structure visible within images. Radiologists learn this structure by viewing thousands of breast images. Once structure is learned, an abnormality can be detected as a location where the tissue looks different than expected. Following this hypothesis, we propose to simulate this phenomenon using a computer algorithm. Specifically, we developed an algorithm that is able to fill in a missing part of an image, at a given location, with what is expected based on the rest of the image and based on what the algorithm has seen in tens of thousands of other images that don’t contain abnormalities. A state-of-the-art generative adversarial network (GAN) is used for this image completion task. A recent study^9^ have shown that image completion algorithms are able to complete images with high-quality patches consistent with their surroundings^9^. Then, if the expected image at this location is different from the actual image, the location is considered suspicious.

The purpose of this research is to determine whether the model trained on data without abnormalities will have difficulty with reconstructing previously unseen abnormal structures. The hypothesis is validated on a set of 70 digital breast tomosynthesis images containing cancerous lesions, by measuring completion error inside and outside of bounding boxes and visualizing model losses in the form of heatmaps.

While GANs have been previously used in the context of anomaly detection^10^, we are familiar with only one study that uses neural network-based image completion for this purpose. Specifically, in a study conducted by Haselmann et al.^11^, mean-squared error (MSE) was incorporated with a GAN to perform image completion (inpainting) abnormality detection, showing promising results but only on a relatively easy task. In this study, the difference between the original image and the completed image (measured by MSE) was used to determine whether a particular location is likely to be abnormal. Here, we extend this study by applying the concept into much more challenging space of medical imaging, introducing a newer attention model for image completion^9^ and evaluating the performance of the model using different mask sizes, model input sizes, and losses on non-trivial medical data.

This study has multiple contributions in terms of its technical aspect and the application. It is the first study that attempts to use image completion for abnormality detection in the context of medical imaging. This comes with a variety of challenges including high resolution images (approximately 50 times more pixels in an image than in^11^. We also introduce the generative image inpainting with contextual attention model^9^ in the context of anomaly detection. Additionally, we use the discrimination loss measure to determine abnormal-looking locations in the context of image completion. Finally, we explore the impact of hyperparameters, such as the field of view and mask size, on the performance of the algorithm.

## Methods

### Dataset

In this study, we used a dataset of digital breast tomosynthesis (DBT) screening studies gathered from the Duke Health System. It contained 4829 studies collected from 4348 patients resulting in 19,230 reconstruction volumes. There are two types of cases within the study: (1) normal and (2) cancer. For the cancer group, lesion bounding boxes were provided by radiologists from Duke Hospital. In the normal group, every study consists of left and right cranial-caudal (CC) and mediolateral-oblique (MLO) views. Studies in the cancer group consist of one or more CC or/and MLO views. Studies with spot compression were not included in our dataset.

The normal set was randomly divided (by patient) into two exclusive training and validation sets with 18,232 and 928 reconstruction views respectively. In addition, we used 70 volumes from the cancer group to evaluate our algorithm in the context of abnormally detection. Six cases where the abnormality was contained within a small distance (128 pixels) from the edge of the image were removed to arrive at the 70 used volumes. From each volume in the normal set (training and validation), we took five random slices/images. From cancer set volumes we only used the slice where radiologist placed a bounding box; if more than one abnormality was marked (which occurred in eight volumes), we selected one slice randomly from the subset of marked slices. The number of cases used for training, validation and testing are shown in Table 1. All data used in this study will be made publicly available on The Cancer Imaging Archive. The retrospective clinical data collection was approved by the Institutional Review Board (IRB) of the Duke University Health System (DUHS), and the methods used in this study were carried out in accordance with relevant guidelines and regulations. The requirement for informed consent was waived by the DUHS IRB.

**Table 1:** Data used during experiments.

Set	Type	Patients	Studies	Volumes	Slices/images
Train	Normal	4109	4558	18,232	91,160
Validation	Normal	200	232	928	4640
Test	Cancer	39	39	70	70

### Generative adversarial networks

**Figure 1:**
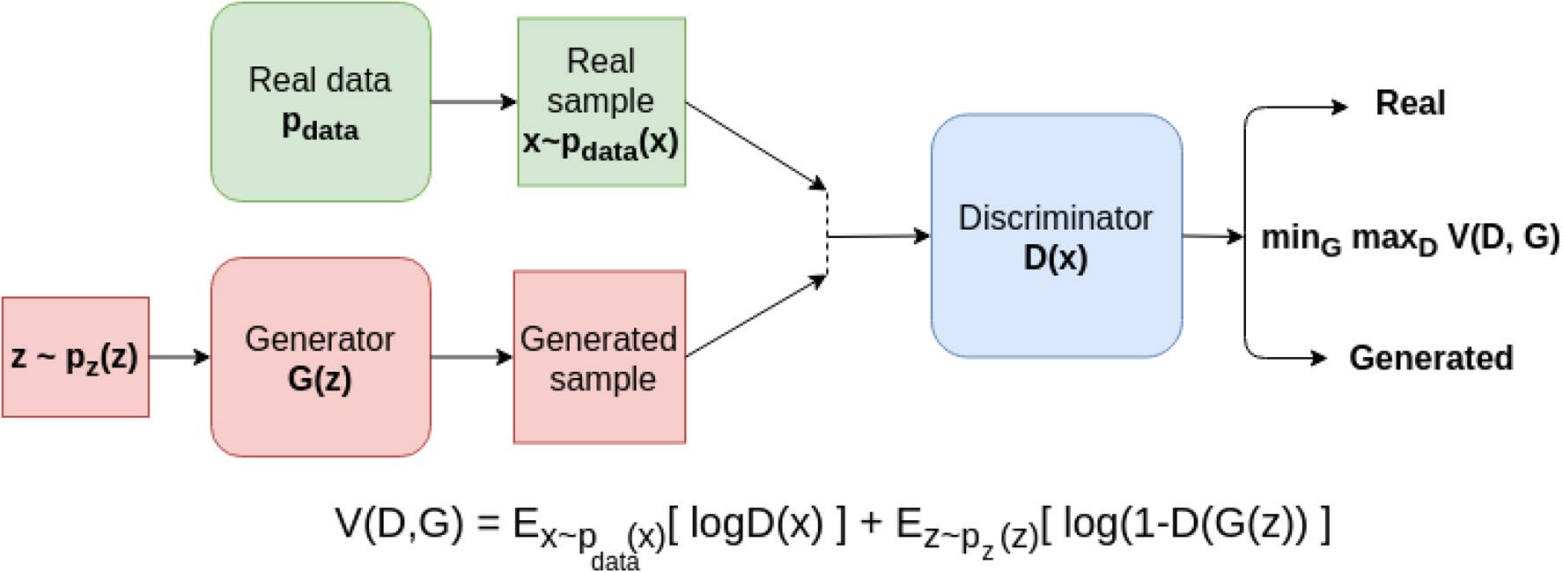
A standard GAN architecture and loss function.

Generative adversarial networks (GANs) introduced in^7^, are based on the idea of two networks competing with each other. One of the networks is responsible for the generation of “fake” training data that appears to be real, by learning to approximate the distribution that generated the real training data. This network is called a generator, denoted $G\left(z\right)$, because it takes a vector of random noise $z$ as input, and maps it to a generated datapoint (image, in our case). The second network, called the *discriminator,* or *critic*, is used to distinguish between generated and true samples. It is labeled $D\left(x\right)$, as the network takes a sample $x$ and outputs the probability of $x$ being from the real dataset. The competition between the two networks can be described as a min–max game of two players trying to beat each other, described in Fig. 1.

**Figure 2:**
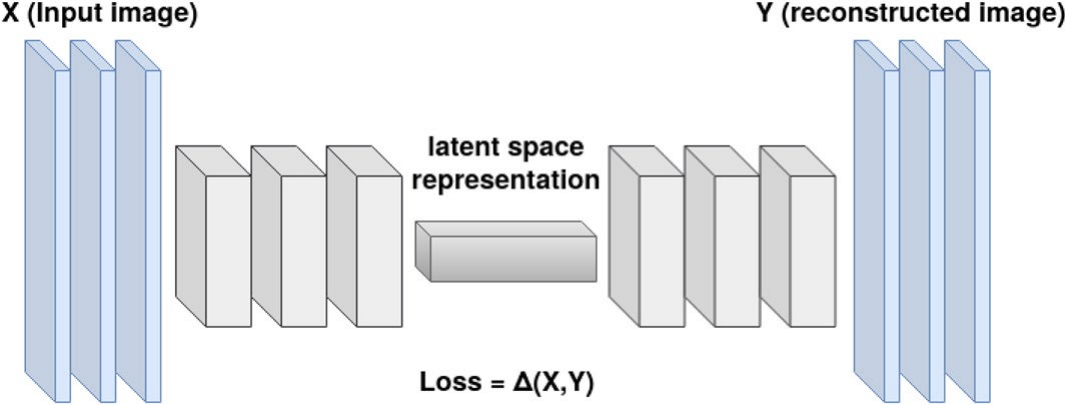
Autoencoder architecture.

GANs with multiple convolutional layers are called deep convolutional GANs (DCGANs)^8^ and are used amongst other methods for the generation of realistic images^12^, image denoising^13^, image translation^14^ and image completion^9^. Image completion is often performed using generators of architecture similar to autoencoders^15^, which foster learning a latent representation of the data. Latent data representation is achieved by compressing and decompressing input data in the way which minimizes information decline. Figure 2 demonstrates sample autoencoder architecture.

### Image completion task and the architecture

In the task of image completion, a part of an image is covered and the model attempts to reconstruct it based on the parts of the image that are present. We assume that the missing part of the image, the *mask*, is square, and we refer to the size of the missing part as the *mask size*. We approached the task of image completion using DCGAN architecture with a two-phase generator followed by local and global discriminators^9^. In order to train the image completion model, we cover part of the image and recreate the covered part using the generator, based on the remainder of the image. We then use the discriminator to estimate the probability of the generated patch being real.

**Figure 3:**
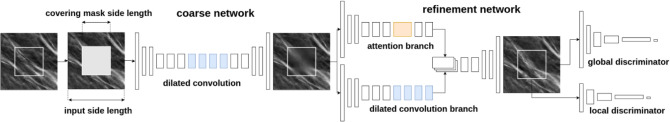
Model architecture and image completion process diagram.

The architecture of the model and a diagram of the image completion process are shown in Fig. 3. In the first stage of the generator, a *coarse* network constructed from dilated convolutional blocks is used to create an imperfect, blurred prediction for the missing patch of the image. The second part of the generator, the *refinement* network, improves the quality of the completed region with more fine-grained details using combined *contextual attention* and dilated convolutional branches. The contextual attention branch, created by^9^, optimizes consistency between the inferred missing patch and the rest of the surrounding image, hereafter the *field of view,* by examining the inner product/cosine-similarity of features within the generated missing patch and features found in the surroundings. Local and global discriminators are responsible for achieving consistency between the completed masked region and the entire image. We experimented with the following parameters: (i) a mask size of 64 × 64 and 128 × 128 pixels, and (ii), a field of view of 256 × 256 and 512 × 512 pixels. To obtain the same dimensionality of feature representation for both of the tested field of view sizes, we append a convolutional layer to the input of the global discriminator module when the field of view size is 512 × 512 pixels.

### Training details

While the min–max objective that dictates the training of GANs (Fig. 1) is conceptually simple, in practice training GANs to give usable results is a difficult task. The goal of generative modeling is essentially to make the “fake” data distribution that the generator learns to sample from, ${P}_{g}$, as similar as possible to the real data distribution ${P}_{r}$. However, if one tries to do this using common distribution divergence/distance metrics, such as the Kullback–Leibler (KL) divergence, that are usually used to train GANs, this optimization procedure is often practically difficult, due to issues such as discontinuities and/or vanishing gradients within the objective function with respect to the network’s parameters, that can occur when a real sample is not within the support of ${P}_{r}$. The Wasserstein distance, described shortly, was proposed as a solution to these problems^16^, and is a key component of our model’s loss function.

The Wasserstein distance between two distributions can intuitively be thought of as the minimal effort needed to transport probability mass between these distributions; it is theoretically defined as

1 $$ \begin{array}{*{20}c} {W\left( {P_{r} ,P_{g} } \right) = {\text{inf}}_{{{\upgamma } \in {\Pi }\left( {P_{r} ,P_{g} } \right)}} E_{{\left( {x,y} \right) \sim {\upgamma }}} \left[ {\left| {\left| {x - y} \right|} \right|} \right],} \\ \end{array} $$

where $\Pi \left({P}_{r},{P}_{g}\right)$ is the set of all distributions $\upgamma$ whose marginal distributions are ${P}_{r}$ and ${P}_{g}$. This equation is unsurprisingly practically intractable, but a more useful form of it can be obtained using the *Kantorovich-Rubinstein duality*^16^, which gives

2 $$ \begin{array}{*{20}c} {W\left( {P_{r} ,P_{g} } \right) = {\text{sup}}_{{f \in {\mathcal{F}}}} E_{{x \sim P_{r} }} \left[ {f\left( x \right)} \right] - E_{{\overline{x} \sim P_{g} }} \left[ {f\left( {\overline{x}} \right)} \right],} \\ \end{array} $$

where $\mathcal{F}$ is the set of all 1-Lipschitz functions. Practically speaking, using $W\left({P}_{r},{P}_{g}\right)$ as the distance measure for training a GAN will modify the min–max objective function (Fig. 1) to become

3 $$ \begin{array}{*{20}c} {\mathop {min}\limits_{G} \mathop {max}\limits_{{D \in {\mathcal{F}}}} E_{{x \sim P_{r} }} \left[ {D\left( x \right)} \right] - {\text{E}}_{{\overline{x} \sim P_{g} }} \left[ {D\left( {\overline{x}} \right)} \right].} \\ \end{array} $$

An important note here is that the discriminator $D$ is constrained to be 1-Lipschitz, which can be thought of as forcing the high-dimensional analog of the “slope” of $D$ with respect to its network parameters to be no greater than 1. Arjovsky et al.^16^ originally implemented this constraint by “clipping” the weights of $D$ to be within a certain magnitude, but this can lead to undesirable training instability. As such, we utilize WGAN-GP in our model, an improved version of the WGAN introduced by^17^ that instead enforces the 1-Lipschitz constraint by adding a *gradient penalty* term

4 $$ \begin{array}{*{20}c} {\lambda E_{{\hat{x} \sim P_{{\hat{x}}} }} \left( {\left| {\left| {\nabla_{{\hat{x}}} D\left( {\hat{x}} \right)} \right|} \right|_{2} - 1} \right)^{2} } \\ \end{array} $$

to the objective function, where $\widehat{x}$ are sampled from the straight line between points sampled from ${P}_{r}$ and ${P}_{g}$ and $\lambda$ is a constant hyperparameter. Essentially what this added term does is instead enforce the aforementioned constraint by penalizing the size of the gradient of $D$ with respect to its input, which gives improved training performance.

We can now write the total loss function ${\mathcal{L}}_{\mathrm{total}}$ for our model as the sum of individual loss components, as

5 $$ \begin{array}{*{20}c} {{\mathcal{L}}_{total} = {\upalpha }_{mask} {\mathcal{L}}_{mask} + {\upalpha }_{FOV} {\mathcal{L}}_{FOV} + {\upalpha }_{GAN} {\mathcal{L}}_{WGAN,G} + {\mathcal{L}}_{WGAN,D} + \lambda {\mathcal{L}}_{WGAN - GP } ,} \\ \end{array} $$

where:

- ${\mathcal{L}}_{mask}$ is the ${L}^{1}$ (Manhattan) distance between the coarse prediction for the masked region and the corresponding region of the ground truth, added to the same for the fine prediction,
- ${\mathcal{L}}_{FOV}$ is the ${L}^{1}$ distance between the coarse prediction for the non-masked part of the image and the corresponding ground truth, added to the same for the fine prediction,
- ${\mathcal{L}}_{WGAN,G}$ and ${\mathcal{L}}_{WGAN,D}$ are the WGAN losses between the local and global discriminators and the two-stage generator (see Eq. 3), with ${\mathcal{L}}_{WGAN-GP}$ being the added gradient penalty (GP) terms for both discriminators (see Eq. 4).

Finally, ${\alpha }_{mask}, {\alpha }_{FOV}$ and ${\alpha }_{GAN}$ are loss weights for each of their respective loss components, and $\uplambda$ is the same WGAN-GP constant of Eq. (4). We set these hyperparameters to the values recommended by Yu et al.^9^ of 1.2, 1.2, 0.001, and 10, respectively. Note that these ${L}^{1}$ losses also utilize the *spatial-discounting* weighting for pixels within the masked region of Yu et al.^9^, where the weight multiplying a given pixel value within the loss is ${0.99}^{l}$, with $l$ being the distance of the given pixel to the nearest *known* pixel outside of the mask. In effect, this is meant to account for the intuitive lesser ambiguity of pixels near the mask boundary than that of pixels near the mask center.

In the training phase, patches of size 256 × 256 or 512 × 512 pixels were sampled randomly from the original images. Then, each patch was covered with a square-shaped mask of pre-determined side length ranging from 16 to 128 pixels. The mask was applied to a random position within the patch field of view. The patch cropping process was conducted in a way that guaranteed overlap of the patches with breast tissue, which was achieved by thresholding non-zero pixels within the random patch choice.

The model was trained with the Adam optimizer^18^ for 2,000,000 iterations and learning rate of 0.0001 with a batch size of 9. The parameters were chosen empirically based on results on the validation set.

### Measuring quality of image completion for a single patch

Once a patch is removed and inpainted by our network, one needs to assess the quality of the replacement. While different metrics could be constructed for this, we relied on two. The first was mean squared error (MSE) and the other was the discriminator loss. The discriminator loss describes the consistency of a filled region within the context of the entire input image according to the discriminator network in the GAN (Fig. 1), but its absolute value cannot be compared between different models, datasets, and stages of the model training process. However, a given model can be applied to different images to assess and compare how much a completed image resembles the data that was used to train it (a normal DBT image in this case). Finally, we also measure the product of the MSE and discriminator loss. We will refer to this metric in the further part of this paper as *DMSE*.

### Identifying abnormalities by measuring image completion quality across entire images

**Figure 4:**
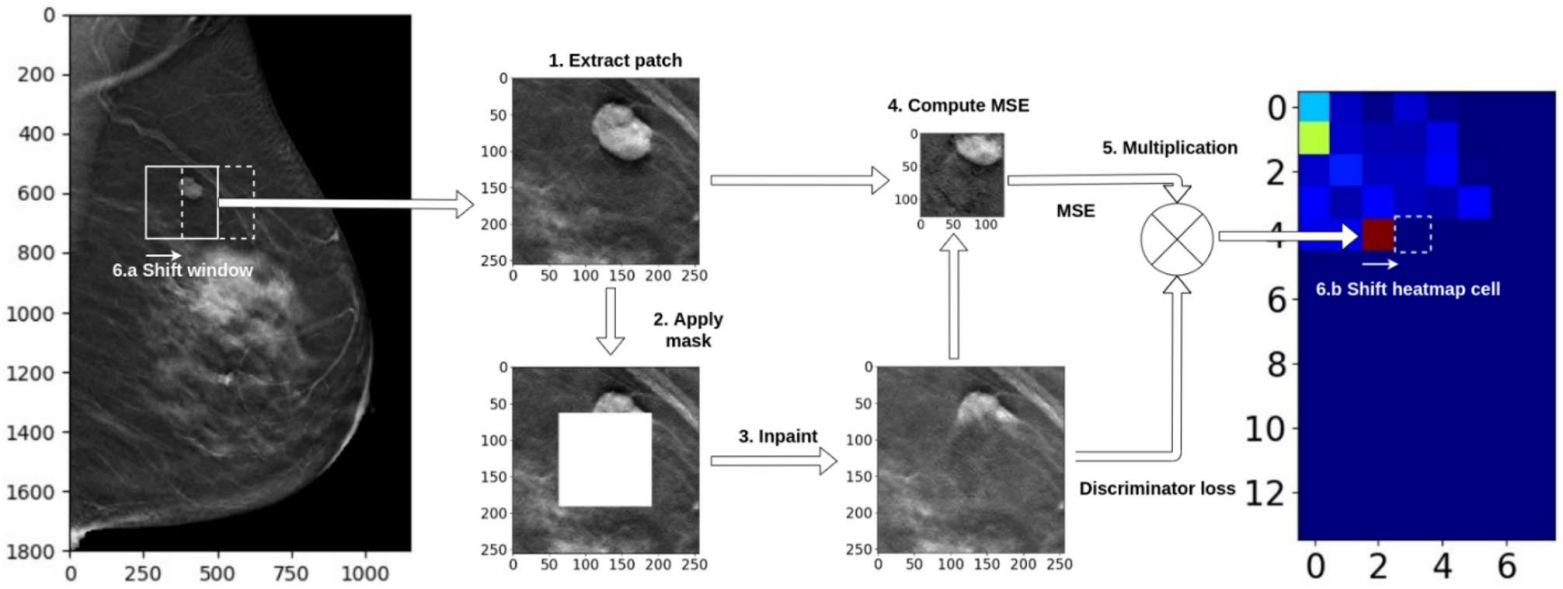
Heatmap generation with sliding window for DMSE metric.

Our hypothesis was that, given some test image, the locations/regions for which our algorithm have more difficulty with correctly completing are more likely to contain an abnormality. As such, to attempt to discover abnormalities within some image, we repeatedly remove parts of the image, inpaint them using our network, and measure the error across the entire image. Specifically, to measure the quality of image completion we used a sliding window approach with a shift value equal to 8 pixels. With every shift we (1) extracted a patch from the original image based on the current position of the window, (2) masked the center part of the extracted patch, (3) generated the missing part of the patch, and (4) measured and saved the computed error metric (MSE, discriminator loss, or DMSE) in a corresponding place on the abnormality heatmap. Figure 4 demonstrates the process of heatmap generation for the DMSE metric.

In addition, we also computed *averaged* heatmaps, described as follows. The process starts with creating a heatmap of the size of the original image, filled with zeros. After computing the loss for some patch, instead of saving it as a single value, we add the loss value to each pixel included within the patch to the corresponding location in the output heatmap. Because the slicing window can cover the same pixels multiple times, the pixels in the final output are divided by the number of times that were included, hence our referring to the heatmaps as “averaged”.

### Evaluation of image completion in the context of detecting abnormalities

After generating heatmaps for every example in the test set, we measured averaged errors for locations inside and outside of radiologist-provided bounding boxes that indicate abnormalities within the set. If our approach is able to discriminate abnormal locations from normal ones, the image completion error will be notably higher for locations which include abnormalities than for normal locations. We consider only pixels inside of breast tissue (excluding background pixels with intensity value equal to zero). Also, since only the middle part of a patch is masked for completion (to ensure sufficient context for the model), areas of the input images close to the edges are not represented in the generated heatmap. The extent of this padding area depends on the field of view and mask size.

We note that although our method was only evaluated on test set images with known lesions, the lesions usually only comprised a small section of each test image. As such, the non-lesion surrounding regions of the test images are just normal breast tissue, and in this way, our algorithm was tested on both normal and cancerous tissue.

## Results

### Image completion

**Figure 5:**
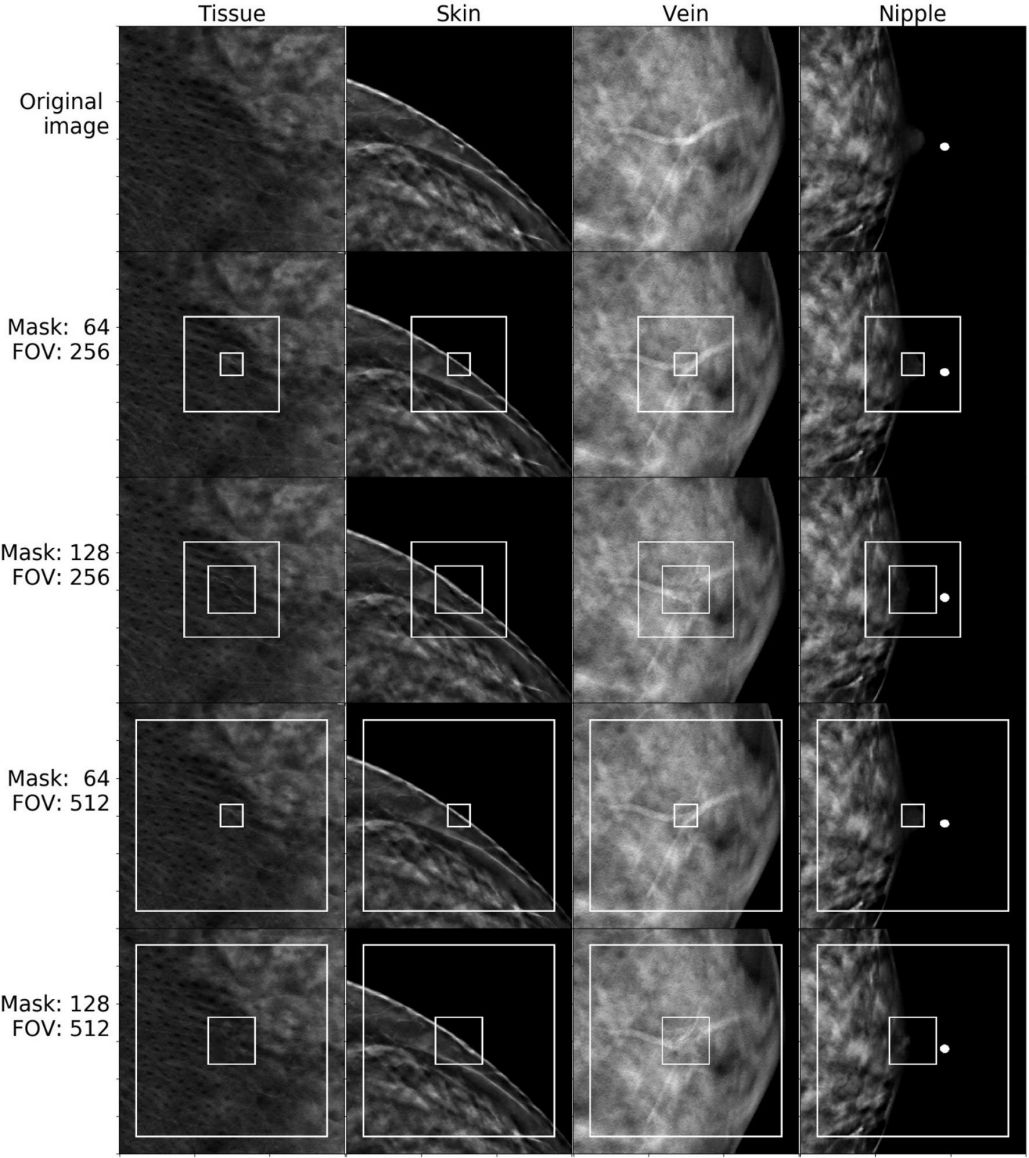
Image completion results for patches containing tissue, breast skin, veins, and nipples.

**Figure 6:**
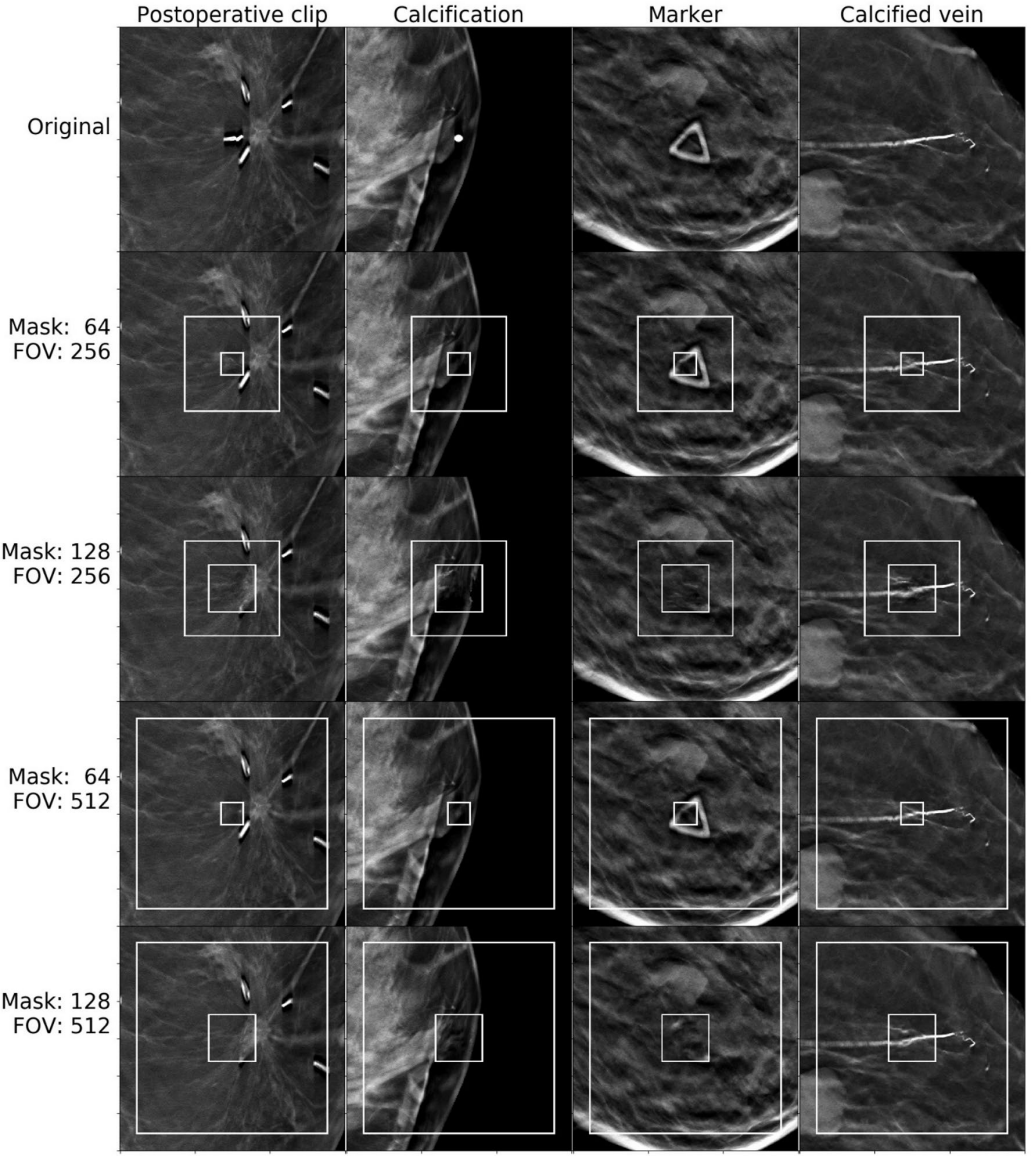
Image completion for patches containing clips, calcification, markers, and calcified veins.

**Figure 7:**
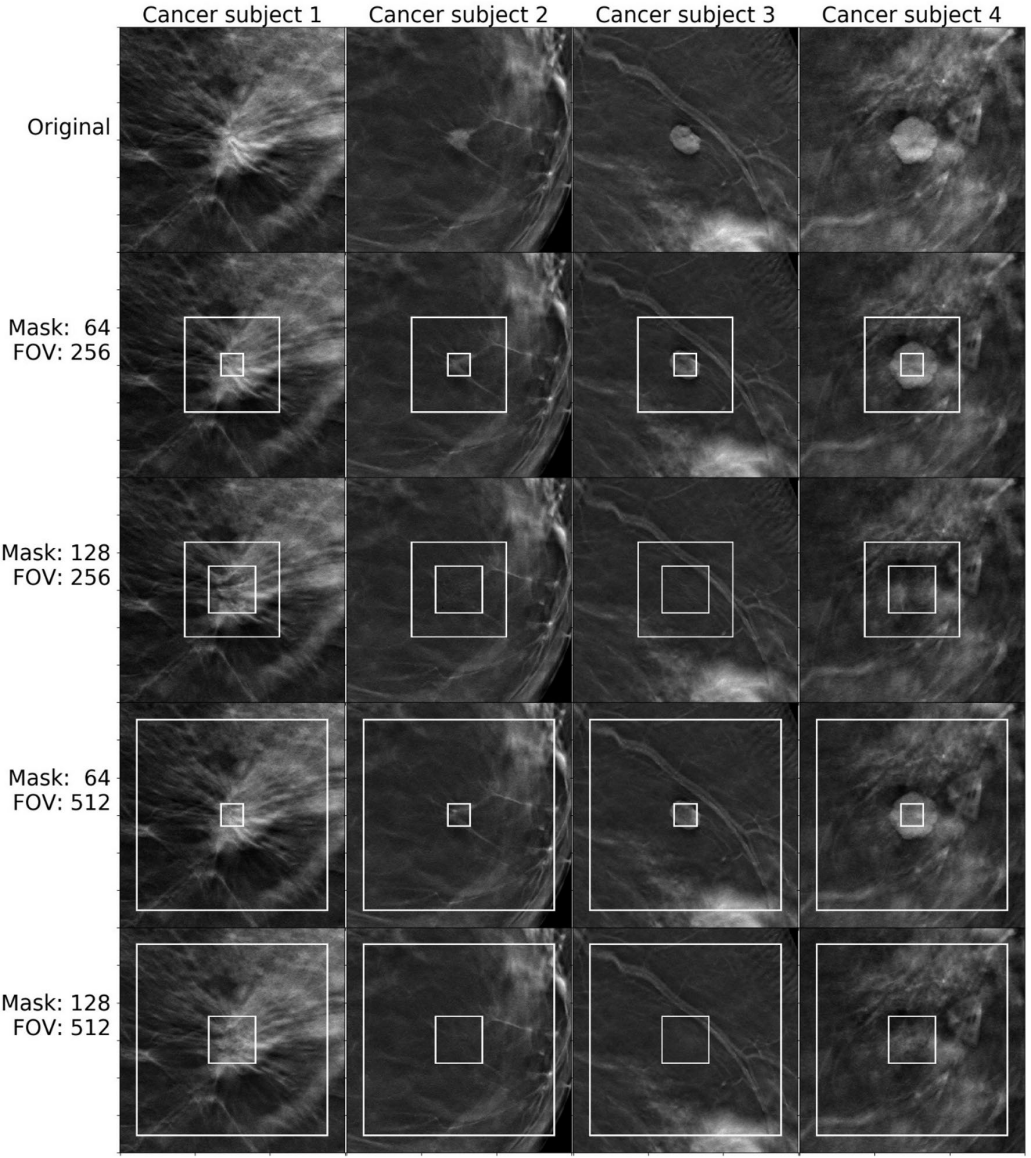
Image completion results for patches containing cancerous masses.

We provide visual references to compare image completion quality between different masks and model input sizes (fields of view, or FOV) for images from the normal set (Fig. 5) and cancer set (Fig. 6 and 7). Masks and fields of view are marked on the images as smaller and larger rectangles respectively. The part of the image covered by the mask was completed based on the remaining part of the FOV patch.

One can see that our approach is capable of generating realistic completions of breast tomosynthesis images including objects such as veins. However, once the removed patch becomes larger, the fidelity of the reconstructed object decreases. In images with unusual objects that are not lesions, when the removed patch fully covers the unusual object, the network did not accurately reconstruct the removed part. As expected, it replaced them with normal-looking tissue (Fig. 6). However, if part of the unusual object (such as a skin marker) was included in the field of view and outside of the removed patch, the network reconstructed the unusual object fairly accurately. This phenomenon was observed for normal and cancer images.

### Abnormality detection

Results for abnormality detection in terms of the mean ratio between heatmap values inside and outside of the ground truth bounding boxes and its standard deviation are given in Table 2. The table shows that the combination of MSE and discriminator loss (DMSE) outperforms the individual metrics, whereas MSE performed better than the discriminator loss. The highest value was obtained for the field of view of 256 × 256 pixels with the mask size of 128 × 128 pixels.

**Table 2:** Ratio of measured losses inside and outside bounding boxes for non-averaged heatmaps; DISCR = discriminator loss, std = standard deviation.

Loss type	Field of view size [pixels]	Mask size [pixels]	Mean ratio [Std]
MSE	256 × 256	64 × 64	1.93 (0.87)
MSE	256 × 256	128 × 128	2.11 (1.01)
MSE	512 × 512	64 × 64	1.83 (1.19)
MSE	512 × 512	128 × 128	1.86 (1.12)
DISCR	256 × 256	64 × 64	1.47 (0.38)
DISCR	256 × 256	128 × 128	1.46 (0.34)
DISCR	512 × 512	64 × 64	1.48 (0.63)
DISCR	512 × 512	128 × 128	1.47 (0.65)
DMSE	256 × 256	64 × 64	2.54 (2.92)
**DMSE**	**256** × **256**	**128** × **128**	**2.77 (1.79)**
DMSE	512 × 512	64 × 64	2.23 (4.33)
DMSE	512 × 512	128 × 128	2.11 (2.68)

**Figure 8:**
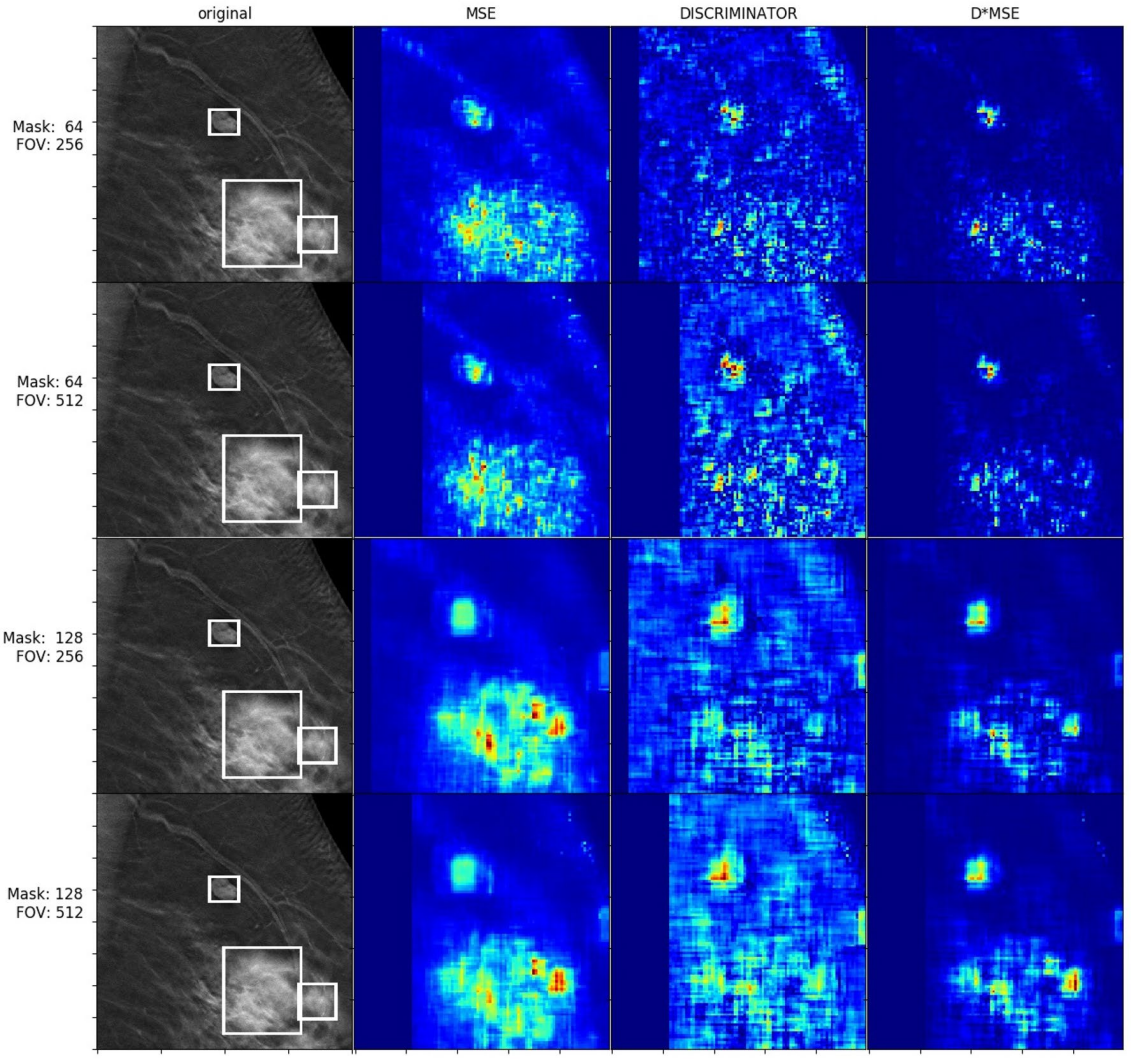
Non-averaged heatmaps for a patient with cancerous masses.

**Figure 9:**
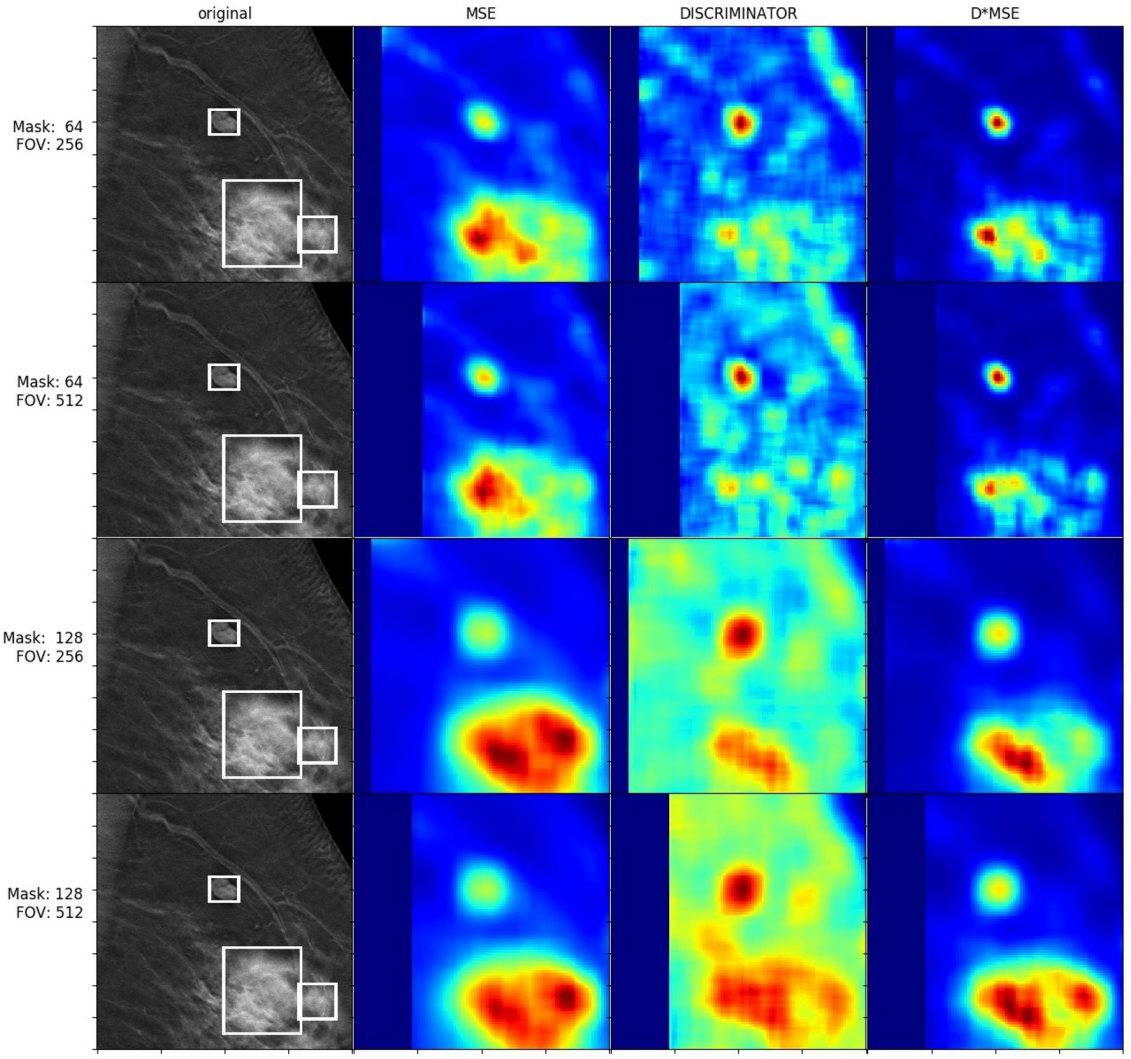
Averaged heatmaps for a patient with cancerous masses.

Figures 8 and 9 contain visualizations of non-averaged and averaged heatmaps, respectively, for the same subject with three separate cancer masses marked by bounding boxes. For the presented examples, the combined error metric diminishes error in areas outside of the bounding boxes as compared to error measures based solely on either MSE or discriminator loss.

## Conclusions and discussion

In this study, we used deep learning-based image completion to identify abnormal locations in digital breast tomosynthesis images. The topic is of high importance because for mammographic cancer detection–as well as many other medical imaging tasks–the availability of abnormal images is very limited and as such, an efficient use of abundantly available normal cases is crucial.

We obtained very realistic results in terms of image completion in DBT images. We showed that the trained model is able to reproduce structures like fibroglandular tissue, skin, and vessels. The covered part was completed with a likely patch from a model based on its surroundings in normal locations and it does not generate unusual objects like postoperative clips or calcifications. When it comes to completing cancerous regions, the results depend on which part of the image in question is masked and completed. If a major part of a cancerous mass or architectural distortion is not covered, the model may reconstruct an abnormality but still produce loss higher than average. We have shown that there is a possibility that this approach can be used in abnormality detection.

In our experiments, we used MSE and discriminator losses in order to describe how well the image was completed. Based on our observations, MSE gives high error values for abnormal objects, e.g. post-operation clips, but also for normal tissue with complex structures such as nipples. On the other hand, we observed that discriminator loss is small while completing all kinds of shapes which were present in the training set, including nipples. Unfortunately, modest values from discriminator loss for completing parts of abnormal images make it difficult to clearly classify tissue as normal or abnormal based on that metric. The results from our study have shown that the combined loss of MSE and discriminator loss worked best. This metric gave high loss value to abnormal patches without being sensitive to sophisticated shapes present in the training set of normal cases.

From the mean ratio values of Table 2 (as well as Figs. 8 and 9), it is clear that the generator cannot inpaint/predict masks over cancerous regions nearly as accurately as that over normal breast tissue. This is to be expected, because the GAN was trained on thousands of scans of normal breast tissue, yet never saw any abnormalities (besides the aforementioned unusual benign objects such as post-operative clips) to learn from and generalize to. Sampling from the tissue image distribution that the generator is attempting to approximate should only result in the generation of normal tissue, so it is unsurprising that the generator has difficulty with synthesizing cancerous tissue, even if the surrounding context of the scan is that of a cancerous breast. Conversely, we see that when our model is used to reconstruct normal regions of breast scans that *do not* include any cancerous tissue/bounding boxes (the majority of the shown test images, as the lesions are small compared to the scale of the entire scans), the DMSE loss (our best-performing metric) is noticeably smaller than in the cancerous regions, on average, showing that normal regions can still be differentiated from cancerous within the output heatmaps. This is because the DMSE loss is proportional to the discriminator loss, which describes how real the discriminator judges the generated tissue to be, compared to the ground truth tissue. Because the discriminator is only trained to discriminate between realistic and non-realistic *normal* tissue, and the generator can only perform poorly and unrealistically when reconstructing regions with abnormalities, this loss is indeed greater for such cancerous regions. As such, the usage of the discriminator provides a further refinement to the loss metrics that are used to indicate abnormalities, explaining why the DMSE metric performed best.

We note that in order to provide classification metrics for our method such as accuracy or ROC (receiver operating characteristic) curves, we would first need to define exactly what a “detection” of cancerous/abnormal tissue is in the context of the generated heatmaps (Figs. 8 and 9). This would require choosing some numerical threshold for the pixel values of the inpainting error/DMSE (Table 2), such that if the error for some pixels/region was greater than this threshold, this region could be described as being detected to be abnormal. In turn, we could compare these error values to known lesion bounding boxes (or use the ratio of error between inside and outside the boxes, as in Table 2) to obtain metrics such as the true positive rate (TPR). However, doing this properly would require further research and experimentation, including questions of the definition of detected regions, overlap criteria, postprocessing and false positive reduction and other questions that we believe are beyond the scope of this work which focuses on the concept of image inpainting.

GANs have proven to be useful in a range of applications, including realistic facial image creation and customization (e.g.^19^, image-to-image translation (e.g.^20^, and even lesser-known applications such as excising rain from images^21^ among others. More particularly related to our method, GAN-based inpainting itself has also seen wide use for a variety of applications, from the conversion of 2D images to 3D representations^22^, to temporally consistent video completion/inpainting^23^, to automatic face-anonymizing for privacy^24^. The use of GANs for abnormality detection is not nearly as common as the aforementioned trend of using GANs for other purposes. However, works such as Herent et al.^25^, Cao et al.^26^, Kooi et al.^27^, Yap et al.^28^ and Yap et al.^29^ also use deep learning for breast lesion detection (e.g., lesion type classification, object recognition and/or segmentation), but they rely on the direct, supervised learning of the appearance of real breast lesions, and as such are distinctly different from our semi-supervised, normal data-based GAN method. Despite this, there is still a group of other generative modeling-based lesion/abnormality detection methods that can be compared to ours.

Benson & Beets-Tan^30^ introduced a method that uses GANs (but with a different inpainting algorithm) to learn the data distribution of normal brain scans and perform inpainting on a grid of masks over the input image, like our method. In this experiment, the sum of the pixel-wise inpainting residuals within each mask are used to indicate abnormalities *mask-by-mask* (if this sum is above a certain numerical threshold),this is essentially the same as our method, just with slightly different masking techniques that create the final outputted abnormality heatmap. Li et al.^31^ proposed a method that is also similar in practice to ours and that of Benson & Beets-Tan, insofar that input test images are divided into mask regions, which are then each separately inpainted one-by-one, after which an anomaly heatmap is generated according to the discrepancy between the original image and the reconstructed image. This model, although originally designed for visual anomaly detection in the context of industrial inspection, is essentially the same idea as our method, with the one difference being that it utilizes encoder-based, rather than the more advanced GAN-based inpainting used in our study.

The advantage of our model is that we do not have to rely on limited cancerous image data. Instead, we train on the abundance of normal scans, a philosophy that certain similar studies share. Chen et al.^13^ also uses an adversarial (but auto-encoder) approach to learning the distribution of healthy *brain* tissue, by learning the mapping of the input scan to some latent space and detecting anomalous scans within this space itself. Schlegl et al.^10^ similarly uses a DCGAN-based architecture (as well as an encoder) to learn the latent space representation and generative process for normal anatomical image data such that at test time, unseen images are mapped to this latent space, and if anomalous, will be noticeably different from their reconstruction, which is found by mapping back from the latent space representation. From here, anomalous regions within the input are detected based on this discrepancy, including both reconstruction and discriminative losses. These methods are similar to ours because of the training on non-anomalous data to learn the distribution for such data. However, there are two main differences when compared to our work. The first is that these works perform image reconstruction using latent representations of data, not with inpainting/direct image completion. The second difference is that at test time, our method performs reconstruction of some masked region using the surrounding non-masked region as input to the network (not viewing the covered region to be reconstructed), while these methods are applied to reconstruct entire images, not patches, of which the networks use the *entire image* as input, not excluding anything to be used in inference, which is distinctly different than our method.

Just as our model does, the two methods of the last paragraph can be used to produce abnormality heatmaps similar to Figs. 8 and 9. However, it is important to note that in the case of the first, auto-encoder-based model, the authors state that the reconstruction quality is predicated on the input image being downsampled to a 32 × 32 resolution. Doing such for our data would drastically reduce the quality of our very high-resolution DBT scan images (even in the training phase, as this uses 256 × 256 inputs), which could produce unforeseen consequences within the training procedure and testing inference. Therefore, in order to compare this method to ours, we would have to use it in a way that it was not intended or downsample our data by a factor of 64 which would dramatically degrade its quality. Similarly, the second method (f-AnoGAN) is built with a DCGAN/WGAN architecture that is designed to have stable training specifically for 64 × 64 images. While this resolution is greater than 32 × 32, either drastically downsampling our input to this resolution, or augmenting the network to accept a larger input, could produce unwanted training issues, or test inference/heatmaps that are not necessarily valid to compare with ours. Alternatively, one could imagine using these methods along a “grid” of disjoint partitions of the input test image, to preserve global test image resolution, but this could potentially result in issues with global cross-partition coherence and consistency. In summary, directly comparing our model to these two models, which are built for lower-resolution images, would likely require considerable further research and development before we obtained results that we are confident in, and as such, this is also beyond the scope of this proof-of-concept work. This reason and the argument outlined in the previous paragraph are the main points for why our method is not immediately reasonable to quantitatively compare to other techniques; in other words, methods with which we can reasonably compare to do not exist.

Our study has certain limitations. First, the number of positive cases in the test set was small. However, it was sufficient to provide a good overview of the algorithm’s performance, on both normal tissue (image regions without legions) and cancerous tissue. Second, the dataset used for evaluation did not contain annotations for all kinds of abnormal objects, e.g. post-operation clips, which did not allow us to provide detailed performance estimation of detection quality. Moreover, the range of tested sizes for the field of view parameter was limited to what we considered reasonable and computationally feasible but larger range of values could be considered in future studies. Another limitation of our approach is that it identified unusual locations in the images that were *not* cancerous. This could be potentially addressed by oversampling such structures during the training. Our approach was also limited by computation time to generate heatmaps since those required thousands of model runs per image. Finally, our no-padding approach leads to omitting boundary parts of the image during detection.

In summary, we showed promising results on how to effectively use data without objects of interest for detection of abnormalities in medical images. Our approach could be further refined via a number of approaches, such as by combining it with fully supervised methods in order to improve performance of object detection with a scarce training signal.
